# Does socio-economic inequality exist in micro-nutrients supplementation among children aged 6–59 months in India? Evidence from National Family Health Survey 2005–06 and 2015–16

**DOI:** 10.1186/s12889-021-10601-6

**Published:** 2021-03-19

**Authors:** Shobhit Srivastava, Shubham Kumar

**Affiliations:** International Institute for Population Sciences, Mumbai, Maharashtra 400088 India

**Keywords:** Vitamin A supplementation, Iron-supplementation, Socio-economic inequality, NFHS, India

## Abstract

**Background:**

Globally, about 25% of children suffer from subclinical vitamin A deficiency (VAD), and approximately 300 million children globally had anemia as per 2011 estimates. Micronutrient deficiencies are generally referred to as “hidden hunger” because these deficiencies developed gradually. The present study determines the socio-economic inequalities in vitamin A supplementation (VAS) and Iron supplementation (IS) among children aged 6–59 months in India and to estimate the change in the percent contribution of different socio-economic correlates for such inequality from 2005 to 06 to 2015–16.

**Methods:**

Data from National Family Health Survey (NFHS) 2005–06 and 2015–16 was used for the analysis. Bivariate analysis and logistic regression analysis was used to carve out the results. Moreover, Wagstaff decomposition analysis was used to find the factors which contributed to explain socio-economic status-related inequality among children in India.

**Results:**

It was revealed that the percentage of children who do not receive vitamin A supplementation was reduced from 85.5% to 42.1%, whereas in the case of IS, the percentage reduced from 95.3% to 73.9% from 2005-06 to 2015–16 respectively. The child’s age, mother’s educational status, birth order, breastfeeding status, place of residence and empowered action group (EAG) status of states were the factors that were significantly associated with vitamin A supplementation and iron supplementation among children in India. Moreover, it was found the children who do not receive vitamin A supplementation and iron supplementation got more concentrated among lower socio-economic strata. A major contribution for explaining the gap for socio-economic status (SES) related inequality was explained by mother’s educational status, household wealth status, and empowered action group status of states for both vitamin A supplementation and iron supplementation among children aged 6–59 months in India.

**Conclusion:**

Schemes like the Integrated Child Development Scheme (ICDS) would play a significant role in reducing the socio-economic status-related gap for micro-nutrient supplementation among children in India. Proper implementation of ICDS will be enough for reducing the gap between rich and poor children regarding micro-nutrient supplementation.

## Background

Globally about 25% of children suffer from subclinical vitamin A deficiency (VAD) [1], and approximately 300 million children globally had anemia as per 2011 estimates [2]. Micronutrient deficiencies are generally referred to as “hidden hunger” because these deficiencies developed gradually [3]. The damages are long run, and devastation is no visible until the irreversible damages have been done [3].

### Iron supplements

Evidence suggests that iron deficiency causes negative effects on cognitive development among children [4]. Moreover, decreased physical capacity and reduced immunity are associated with iron deficiency among children [5]. Reduced immunity causes children to be at higher susceptibility to infectious diseases [6]; additionally, iron deficiency causes retarded growth of pre-school and school-going children’s [6]**.** Children from lower socio-economic status (SES) had higher iron deficiency than children from higher SES [7]. Previous studies found a significant association between iron deficiency among children and breastfeeding status [8]. The educational status of parents and the wealth status of the family are strong correlates of iron deficiency among children [9]. Further birth order of children was carved out to be a strong predictor of iron supplementation among children [5]. Moreover, the study found that there was high spatial autocorrelation of anemia among children in India, i.e. anemia was highly correlated with spatial factors [10]. Additionally, iron deficiency (ID) mostly resulting in anemia which was very prevalent among children throughout the world, especially in developing countries [11]. The higher prevalence of anemia among children in developing countries can be attributed to low intake of iron supplements in the early years of life which is known for the fast development of children’s lifestyle, geographical factors, socio-economic status and children’s nutritional status [12].

### Vitamin A supplements

It was concluded in previous studies that vitamin A deficiency was magnified by poverty status and higher prevalence of infectious diseases [13], and VAD is the underlying cause of measles, diarrhea and malaria globally [13]. Additionally, VAD causes night blindness problem, Bitot’s spots and other morbid conditions [14]. Further, VAD was positively associated with stunting and wasting among children [15]. Interestingly, it was too argued that VAD was highly correlated with the development of anemia because it plays an important role in red blood cell production and iron metabolism [14]. Vitamin A deficiency was strongly associated with maternal educational level [16], breastfeeding practices [16], being a male child [17], a child age 48–59 months [17] and children from poor socio-economic status [16]. Moreover, children from lower levels of social and economic development and higher birth orders had a 50% lesser likelihood to receive vitamin A supplements [16].

### Children dietary patterns in relation to VAD and ID

Awareness of important dietary intakes influences the nutritional status of infant and young child. As the World Health Organization (WHO) has already cited that, iron deficiency (ID) was the most prevalent nutritional deficiency [18, 19], whereas a different study reported that vitamin A deficiency (VAD) was a common form of micronutrient malnutrition that affecting 21% of pre-school age children worldwide [20]. Micronutrient deficiencies were mainly associated with inadequate dietary intake, increased losses from the body, and increased requirements [21]. A systematic review indicated that VAD leads to serious health problems among pre-school age children, which were mainly due to high prevalence of morbidity, poor consumption of fruits and vegetables, the monotonous cereal-legume diet, poor consumption of vitamin A diet and lower vitamin A supplementation [22].

Micronutrients are acknowledged as an important component in public health. Such micronutrients like zinc, iron, selenium, copper, vitamin A, E, C, D, B2, B6, B12 and folate are necessary for the human body, especially among children [23]. Although the implementation of many micronutrient supplementation programs, very few countries have achieved the targets and have undertaken comprehensive surveys on micronutrient deficiencies [24]. In last, there is a need for a comprehensive micronutrient implementation program with a focus on multiple micronutrients altogether.

There was existing literature that focuses on the determinants of micro-nutrients supplementation among children aged 6–59 months in India. Various literature found the important correlates of VAS and IS among children aged 6–59 months in India. However, there seems a scarcity of studies that aimed to focus on the change in socio-economic related inequality for VAS and IS over the period of time in India. Therefore the present study aims to determine the extent of socio-economic inequality which exist in VAS and IS among children aged 6–59 months in India and to estimate the change in the percent contribution of different socio-economic correlates for such inequality from 2005-06 to 2015–16. The study hypothesized that there was no change in socio-economic inequality in terms of VAS and IS among children aged 6–59 months in India from 2005 to 06 to 2015–16.

## Methods

### Data

The study used data from two rounds of the National Family Health Survey (NFHS) as NFHS-3 and NFHS-4 conducted in 2005–06 and 2015–16, respectively. The nodal agency for conducting these surveys is the International Institute for Population Sciences, Mumbai. The NFHS-3 and NFHS-4 are national representatives; the cross-sectional survey used a systematic, two-stage, cluster sample of the household. The survey provides information on several new and emerging issues, including family planning, nutrition, education, adolescent reproductive health and morbidities. The women’s questionnaire collected all eligible women aged 15–49 years in both rounds of the survey. We included 46,890 samples of children for 2005–06 and 236,977 children for 2015–16 aged 6–23 months [25].

### Dependent variable

The dependent variable for this study was vitamin A and iron supplementation among children aged 6–59 months. The response was recorded by asking the question, ‘whether vitamin A dose was given in the last six months? The responses were recorded as either ‘yes’ or ‘no’. Likewise, the question on iron supplements was asked ‘in the last 7 days, was given iron pills or iron syrup? The response was recorded as 0 “yes” and 1 “no”. The children who did not receive the supplementations were considered to be deficient in vitamin A and iron.

### Covariates

The covariates included in the analysis are children’s age in months was coded as 6–23, 24–59. The sex of the child was coded male and female. Mother’s educational status was coded as not educated, primary, secondary and higher. Birth order was coded as 1, 2–3 and 4–6. Current breastfeeding status was coded as yes and no. The wealth index was coded poor, middle and rich. The variable of wealth status was created using the information given in the survey. Households were given scores based on the number and kinds of consumer goods they own, ranging from a television to a bicycle or car, and housing characteristics such as the source of drinking water, toilet facilities, and flooring materials. These scores are derived using principal component analysis. National wealth quintiles are compiled by assigning the household score to each usual (de jure) household member, ranking each person in the household population by their score, and then dividing the distribution into three equal categories [25]. Caste was coded as SC/ST, and non-SC/ST includes OBC and others. The Scheduled Caste include “untouchables”, a group of the population that is socially segregated and financially/economically by their low status as per Hindu caste hierarchy. The Scheduled Castes (SCs) and Scheduled Tribes (STs) are among the most disadvantaged socio-economic groups in India. The OBC are the group of people who were identified as “educationally, economically and socially backwards”. The OBC’s are considered low in the traditional caste hierarchy but are not considered untouchables. The “other” caste category are identified as having higher social status [26]. Religion was coded as Hindu, Muslim and others. Other religious category included Jain/Sikh/Jewish/Buddhist/others. Place of residence was coded as rural and urban, and the states of India were grouped as Empowered Action Group (EAG) states and non-EAG states [27]. The Indian states were classified into EAG and Non-EAG regions based on key development indicators such that the states belonging to the EAG region significantly lag behind the states in the Non-EAG region on the basis of key human development indicators. The EAG region includes the eight Indian states of Bihar, Chhattisgarh, Jharkhand, Madhya Pradesh, Odisha, Rajasthan, Uttaranchal, and Uttar Pradesh, whereas all the other remaining Indian states comprise the Non-EAG region.

### Statistical analysis

Bivariate analysis was carried out to estimate the prevalence of children who do not receive vitamin A supplementation (VAS) and iron supplementation (IS). Two sample proportion test was used to find whether the difference for VAS and IS between two time period (2005–06 and 2015–16) was significant or not. Additionally, binary logistic regression analysis was used to establish the association between the dependent variable with the covariates [28]. To estimate socio-economic inequalities for VAS and IS, a concentration index, concentration curve and decomposition analysis was employed, which represents the degree of inequality.

The concentration curve is obtained by plotting the cumulative proportion of outcome variables (VAS and IS) on the y-axis against the increasing percentage of the population ranked by the socio-economic indicator (wealth index) on the x-axis. The curves show that whether the socio-economic status-related inequality in the outcome variable (on the x-axis) prevails or not. If the curve is above the line of equality (45-degree line), that means the index value is negative; hence it shows that the outcome variable is disproportionally concentrated among the poor and vice-versa. Income-related inequality in VAS and IS was measured by the concentration index (CI) and the concentration curve (CC), using the wealth score as the socio-economic indicator and binary outcome as VAS and IS. The concentration index is defined as twice the area between the concentration curve and the line of equality. The concentration index measures the inequality of one variable (say VAS and IS) over the distribution of another variable (wealth index). The index ranges from − 1 to + 1, where the index value of 0 (zero) shows no socio-economic inequality. However, the positive value of the index shows pro-rich inequality and vice-versa. Additional, on either scales higher the value, the higher the extent of socio-economic inequality. The study used Wagstaff decomposition analysis to decompose the concentration index. Wagstaff’s decomposition demonstrated that the concentration index could be decomposed into the contributions of each factor to the income-related inequalities [29]. For any linear regression model on a health outcome (y) (say VAS and IS), such as

1 $$ y=\alpha +{\sum}_k{\beta}_k{x}_k+\varepsilon $$

The concentration index for y, C, can be written as follows,

2 $$ C={\sum}_k\left({\beta}_k{\overline{x}}_k/\mu \right){C}_k+G{C}_{\varepsilon }/\mu $$

Where *μ* is the mean of y, ${\overline{x}}_k$ is the mean of *x*_*k*_, *C*_*k*_ is the concentration index for *x*_*k*_ (defined analogously to C), and *GC*_*ε*_ is the generalized concentration index for the error term (*ε*). Eq. (2) shows that C is equal to a weighted sum of the concentration indices of the k regressor, where the weight for *x*_*k*_ is the elasticity of y with respect to *x*_*k*_ $\left({\eta}_k={\beta}_k\frac{{\overline{x}}_k}{\mu}\right)$. The residual component captured by the last term reflects the socio-economic status-related inequality in health that is not explained by systematic variation in the regressor by income, which should approach zero for a well-specified model. Each contribution is the product of elasticity with the degree of economic inequality. Moreover, the percentage contribution is obtained by dividing each absolute contribution by total absolute contribution multiplied by 100 to obtain the estimates [30].

## Results

Table 1 revealed that the children who did not receive VAS were significantly reduced from 85.5% to 42.1%, whereas the children who did not receive IS were significantly reduced from 95.3% to 73.9% from 2005 to 06 to 2015–16, respectively.

**Table 1:** Socio-economic characteristics of children aged 6–59 months in India, 2005–06 and 2015–16

Background characteristics	Categories	2005–06		2015–16	
		n	%	n	%
**Vitamin A supplementation**	No	6379	14.5	1,30,252	57.9
	Yes	37,634	85.5	94,841	42.1
**Iron supplementation**	No	2053	4.7	58,686	26.1
	Yes	41,961	95.3	1,66,406	73.9
**Age of the child**	6–23 months	14,422	33.2	74,132	33.4
	24–59 months	28,997	66.8	1,47,726	66.6
**Sex of the child**	Male	24,487	52.0	1,23,433	52.0
	Female	22,403	47.8	1,13,544	47.9
**Mother’s education**	No education	19,230	41.0	75,010	32.0
	Primary	6777	14.5	34,790	15.0
	Secondary	17,346	37.0	1,05,722	45.0
	Higher	3536	7.5	21,455	9.0
**Birth order**	1st order	32,220	68.7	1,68,425	71.0
	2–3 order	14,562	31.1	68,099	29.0
	4–6 order	108	0.2	453	0.0
**Currently breastfeeding**	No	18,018	38.4	90,797	38.0
	Yes	28,872	61.6	1,46,180	62.0
**Wealth index**	Poor	17,021	36.0	1,18,786	50.0
	Middle	9697	21.0	47,265	20.0
	Rich	20,172	43.0	70,926	29.9
**Caste**	SC/ST	44,673	96.6	92,221	41.0
	Non SC/ST	1594	3.5	1,34,150	59.0
**Religion**	Hindu	32,273	69.9	1,71,115	73.0
	Muslim	7818	16.9	37,412	16.0
	Others	6066	13.1	25,607	10.9
**Place of residence**	Urban	17,782	37.9	56,298	24.0
	Rural	29,108	62.1	1,80,679	76.0
**EAG and non-EAG States**	EAG and Assam	8776	18.7	1,43,219	60.4
	Non-EAG	38,114	81.3	93,758	39.6

*EAG states include Bihar, Jharkhand, Uttar Pradesh, Uttarakhand, Madhya Pradesh, Chhattisgarh, Orrisa, Rajasthan and Assam

*Non-EAG states include rest of Indian states except EAG and Assam

*SC/ST* Scheduled Caste/Scheduled Tribe

Table 2 represents the percentage of children aged 6–59 months who did not receive VAS and IS by their background characteristics in India. The percentage of children who did not receive VAS was higher in the age group 24–59 months. However, the percentage of children who did not receive IS were higher in the age group 24–59 months in 2005–06 and 6–23 months in 2015–16. The percentage of children who did not receive VAS and IS were higher if the mother was not educated. Additionally, children from higher birth order had a higher share for not receiving VAS and IS. The percentage of children who did not receive VAS and IS were higher in the poor wealth status category and rural residential status.

**Table 2:** Bivariate association for children who did not receive VAS and IS among children aged 6–59 months by background characteristics in India, 2005–06 and 2015–16

Background characteristics	Categories	Children who did not receive VAS		Difference (*p*-value)	Children who did not receive IS		Difference (*p*-value)
		2005–06	2015–16		2005–06	2015–16	
**Age of the child**	6–23 months	79.1	40.0	39.1 (0.001)	95.0	74.2	20.8 (0.001)
	24–59 months	88.5	43.1	45.4 (0.001)	95.5	73.9	21.6 (0.001)
**Sex of the child**	Male	85.5	42.1	43.4 (0.001)	95.0	73.8	21.2 (0.001)
	Female	85.5	42.2	43.3 (0.001)	95.6	74.1	21.5 (0.001)
**Mother’s education**	No education	89.4	50.6	38.8 (0.001)	97.4	79.1	18.3 (0.001)
	Primary	84.9	44.8	40.1 (0.001)	95.5	75.5	20.0 (0.001)
	Secondary	80.5	37.3	43.2 (0.001)	93.1	71.0	22.1 (0.001)
	Higher	80.6	35.3	45.3 (0.001)	88.6	69.7	18.9 (0.001)
**Birth order**	1st order	83.4	40.7	42.7 (0.001)	94.9	73.3	21.6 (0.001)
	2–3 order	90.2	46.0	44.2 (0.001)	96.3	75.5	20.8 (0.001)
	4–6 order	91.6	60.7	30.9 (0.001)	98.8	83.5	15.3 (0.001)
**Currently breastfeeding**	No	87.4	41.9	45.5 (0.001)	94.1	72.5	21.6 (0.001)
	Yes	84.6	42.3	42.3 (0.001)	96.0	74.8	21.2 (0.001)
**Wealth index**	Poor	87.5	46.7	40.8 (0.001)	97.2	77.4	19.8 (0.001)
	Middle	84.6	40.2	44.4 (0.001)	95.7	72.6	23.1 (0.001)
	Rich	83.2	37.0	46.2 (0.001)	92.5	69.9	22.6 (0.001)
**Caste**	SC/ST	85.6	41.9	43.7 (0.001)	95.3	73.2	22.1 (0.001)
	Non SC/ST	84.0	42.4	41.6 (0.001)	95.8	74.6	21.2 (0.001)
**Religion**	Hindu	85.5	41.2	44.3 (0.001)	95.3	73.6	21.7 (0.001)
	Muslim	85.6	47.9	37.7 (0.001)	96.1	76.8	19.3 (0.001)
	Others	85.1	35.4	49.7 (0.001)	93.7	68.2	25.5 (0.001)
**Place of residence**	Urban	84.5	39.2	45.3 (0.001)	93.0	70.9	22.1 (0.001)
	Rural	85.9	43.3	42.6 (0.001)	96.2	75.2	21.0 (0.001)
**EAG and non-EAG States**	EAG and Assam	78.0	50.3	27.7 (0.001)	91.8	80.7	11.1 (0.001)
	Non-EAG	87.1	32.5	54.6 (0.001)	96.1	65.9	30.2 (0.001)

EAG states include Bihar, Jharkhand, Uttar Pradesh, Uttarakhand, Madhya Pradesh, Chhattisgarh, Orissa, Rajasthan and Assam

*Non-EAG states include the rest of Indian states except EAG and Assam

*Differences* 2005–06 – 2015-16, *VAS* Vitamin A supplementation, *IS* Iron supplement supplementation, *SC/ST* Scheduled Caste/Scheduled Tribe

Table 3 presents the odds ratio for children who did not receive VAS and IS by their background characteristics in India. The odds for not receiving VAS was found higher among children aged 24–59 months when compared to children aged 6–23 months in 2005–06 (OR: 1.85) and 2015–16 (OR: 1.08). Children whose mother had a higher level of education had significantly lower odds for not receiving VAS compared with mother’s with no education in 2005–06 (OR: 0.50) and 2015–16 (OR: 0.69). The results revealed that the odds for not receiving VAS was higher among children from higher birth order in 2005–06 and 2015–16. Additionally, the children whose mothers breastfeed had lower odds for not receiving VAS than mother’s who did not breastfeed in 2005–06 (OR: 0.80) and 2015–16 (OR: 0.90). In 2005–06 the odds for not receiving iron supplementation among children was higher in non-EAG states in reference to EAG states (OR: 1.74); however, in 2015–16, the association got inversed (OR: 0.50).

**Table 3:** Logistic regression estimates for children who did not receive VAS and IS among children aged 6–59 months by background characteristics in India, 2005–06 and 2015–16

Background characteristics	Categories	Children who did not receive VAS		Children who did not receive IS	
		2005–06 (OR)	2015–16 (OR)	2005–06 (OR)	2015–16 (OR)
**Age of the child**	6–23 months	Ref.	Ref.	Ref.	Ref.
	24–59 months	1.85***	1.08***	1.03	0.96**
**Sex of the child**	Male	Ref.	Ref.	Ref.	Ref.
	Female	0.98	0.99	1.11	1.02
**Mother’s education**	No education	Ref.	Ref.	Ref.	Ref.
	Primary	0.71***	0.88***	0.68***	0.92*
	Secondary	0.51***	0.73***	0.53***	0.85***
	Higher	0.50***	0.69***	0.36***	0.82***
**Birth order**	1st order	Ref.	Ref.	Ref.	Ref.
	2–3 order	1.28***	1.09***	1.15**	1.03
	4–6 order	1.22	1.53*	2.48	1.43
**Currently breastfeeding**	No	Ref.	Ref.	Ref.	Ref.
	Yes	0.80***	0.90***	1.14**	0.99
**Wealth index**	Poor	Ref.	Ref.	Ref.	Ref.
	Middle	0.94	1.02	0.81**	0.98
	Rich	1.00	0.99	0.59***	0.95
**Caste**	SC/ST	Ref.	Ref.	Ref.	Ref.
	Non SC/ST	1.14	1.01	1.21	1.08***
**Religion**	Hindu	Ref.	Ref.	Ref.	Ref.
	Muslim	0.94	1.27***	1.07	1.16***
	Others	1.09	1.11*	0.94	1.10***
**Place of residence**	Urban	Ref.	Ref.	Ref.	Ref.
	Rural	0.84***	0.90***	1.08	0.97
**EAG and non-EAG States**	EAG and Assam	Ref.	Ref.	Ref.	Ref.
	Non-EAG	1.74***	0.50***	1.97***	0.48***

Note- Significant at *p < 0.10, **p < 0.005, ***p < 0.01; *OR* Odds Ratio

Ref- Reference category

EAG states include Bihar, Jharkhand, Uttar Pradesh, Uttarakhand, Madhya Pradesh, Chhattisgarh, Orissa, Rajasthan and Assam

*Non-EAG states include rest of Indian states except EAG and Assam

*VAS* Vitamin A supplementation, *IS* Iron supplement supplementation, *SC/ST* Scheduled Caste/Scheduled Tribe

In 2015–16 the children from the age group 24–59 months had a lower likelihood for not receiving IS than children from age group 6–23 months. Children whose mother had a higher level of education had significantly lower odds for not receiving IS compared with mother’s with no education in 2005–06 (OR: 0.36) and 2015–16 (OR: 0.82), respectively. The results revealed that the odds for not receiving IS was higher among children from higher birth order in 2005–06 and 2015–16. In 2005–06 children from the rich wealth quintile had lower odds for not receiving IS in reference to children from the poor wealth quintile. In 2005–06 the odds for not receiving iron supplementation among children was higher in non-EAG states in reference to EAG states (OR: 1.97); however, in 2015–16, the association got inversed (OR: 0.48).

**Fig. 1:**
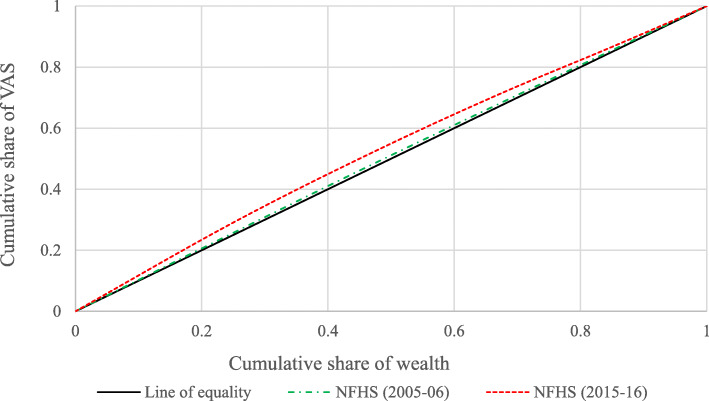
Change in concentration curve for children who did not receive VAS in India, 2005–06 and 2015–16

**Fig. 2:**
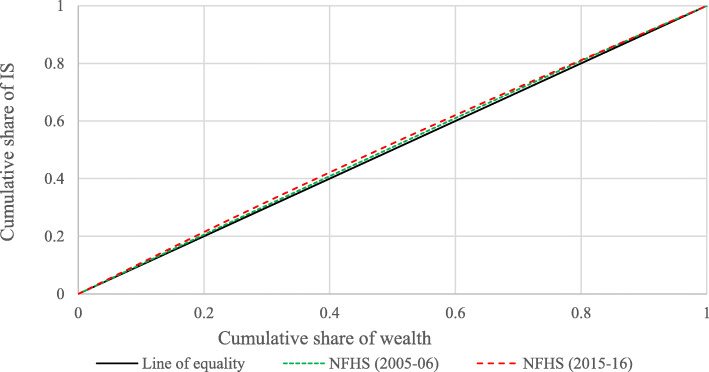
Change in concentration curve for children who did not receive IS in India, 2005–06 and 2015–16

Figures 1 and 2 show the concentration curve for VAS and IS in 2005–06 and 2015–16. It shows the extent of socio-economic inequality among children who did not receive micronutrients supplementation (VAS & IS). The concentration curve for both rounds lies above the 45-degree diagonal line that indicates a higher level of non-recipient of micronutrient supplementation (VAS & IS) concentrated among children from poor socio-economic status. The curve in 2015–16 for VAS and IS got higher than the curve for 2005–06, indicating increasing socio-economic status-related inequality among children aged 6–59 months.

Table 4 reveals decomposition analysis for the contribution of the various explanatory variable for VAS among children age 5–59 months from 2005 to 06 to 2015–16. The table contains information about coefficient, elasticity, CI, absolute contribution to CI and percent contribution. The value of absolute contribution indicates the extent of inequality contributed by the explanatory variables. The value of negative sign in CI indicates the more concentration of VAS among the poor, where a positive value indicates concentration among the rich for the same. Mother’s educational status explained a major part of SES related inequality for children who did not receive VAS in 2005–06; however, in 2015–16, the contribution declined significantly. Inversely, in 2005–06 the EAG and non-EAG status of the Indian states explained only about 7.9% of SES related inequality, whereas, in 2015–16, the contribution increased to 63.7%. Additionally, the wealth index explained about 7% of SES related inequality for children who did not receive VAS in 2005–06 and 2015–16.

**Table 4:** Estimates of decomposition analysis for the contribution of various explanatory variables for children who did not receive VAS among children aged 6–59 months by background characteristics in India, 2005–06 and 2015–16

Background characteristics	Categories	2005–06					2015–16				
		Coefficient	Elasticity	CI	Absolute contribution to CI	Percentage contribution	Coefficient	Elasticity	CI	Absolute contribution to CI	Percentage contribution
**Age of the child (months)**	06–23	Ref.					Ref.				
	24–59	0.514	0.047	−0.005	0.000	1.9	0.030	0.004	− 0.002	0.000	0.0
**Sex of the child**	Male	Ref.					Ref.				
	Female	−0.020	− 0.001	− 0.009	0.000	0.0	− 0.007	− 0.002	− 0.01	0.000	− 0.1
**Mother’s education**	No education	Ref.					Ref.				
	Primary	−0.336	− 0.005	−0.02	0.000	−0.8	− 0.118	−0.004	− 0.169	0.001	−2.6
	Secondary	−0.679	−0.026	0.375	−0.010	79.8	−0.303	− 0.033	0.187	− 0.006	23.6
	Higher	−0.709	−0.004	0.804	−0.003	28.9	−0.371	− 0.009	0.642	− 0.006	22.3
**Birth order**	1st order	Ref.					Ref.				
	2–3 order	0.332	0.011	−0.076	− 0.001	6.9	0.133	0.008	−0.110	− 0.001	3.5
	4–6 order	0.292	0.000	−0.239	0.000	0.2	0.462	0	−0.301	0.000	0.2
**Currently breastfeeding**	No	Ref.					Ref.				
	Yes	−0.220	− 0.017	−0.082	0.001	−11.4	− 0.104	− 0.016	− 0.081	0.001	−4.9
**Wealth index**	Poor	Ref.					Ref.				
	Middle	− 0.075	−0.002	0.155	0.000	2.5	0.010	−0.001	0.145	0.000	0.6
	Rich	−0.026	−0.001	0.676	−0.001	4.8	−0.020	− 0.003	0.672	− 0.002	6.5
**Caste**	SC/ST	Ref.					Ref.				
	Non SC/ST	0.164	0.001	−0.077	0.000	0.2	0.009	−0.002	0.100	0.000	0.6
**Religion**	Hindu	Ref.					Ref.				
	Muslim	−0.074	−0.002	0.003	0.000	0.0	0.238	0.011	0.022	0.000	−1.0
	Others	0.076	0.000	0.308	0.000	−1.0	0.109	0.001	0.302	0.000	−1.5
**Place of residence**	Urban	1.000					1.000				
	Rural	−0.150	−0.014	−0.168	0.002	−19.8	−0.092	− 0.016	−0.181	0.003	−10.8
**EAG and non-EAG**	EAG and Assam	1.000					1.000				
	Non-EAG	0.567	0.066	−0.015	−0.001	7.9	−0.684	− 0.074	0.222	− 0.016	63.7
Explained CI					−0.012	100.0				−0.026	100.0
Actual CI					−0.014					−0.065	
Residuals					−0.002					−0.039	

EAG states include Bihar, Jharkhand, Uttar Pradesh, Uttarakhand, Madhya Pradesh, Chhattisgarh, Orissa, Rajasthan and Assam

*Non-EAG states include rest of Indian states except EAG and Assam

*CI* concentration index, *Ref* Reference, *EAG* empowered action group, *VAS* Vitamin A supplementation, *SC/ST* Scheduled Caste/Scheduled Tribe

Table 5 reveals decomposition analysis for the contribution of the various explanatory variable for IS among children age 5–59 months from 2005 to 06 to 2015–16. Mother’s educational status explained about 45% of SES related inequality for children who did not receive IS in 2005–06; however, in 2015–16, the contribution declined by almost half the contribution in 2005–06. Even, wealth index explained 42% of SES related inequality for children who did not receive IS in 2005–06; however, in 2015–16, the contribution declined by 18.8 points to 23.4% in 2015–16. In 2005–06 EAG and non-EAG status of the Indian states explained only about 4.1% of SES related inequality for children who did not receive IS, whereas, in 2015–16, the contribution increased to 70.8%.

**Table 5:** Estimates of decomposition analysis for the contribution of various explanatory variables for children who did not receive IS among children aged 6–59 months by background characteristics in India, 2005–06 and 2015–16

Background characteristics	Categories	2005–06					2015–16				
		Coefficient	Elasticity	CI	Absolute contribution to CI	Percentage contribution	Coefficient	Elasticity	CI	Absolute contribution to CI	Percentage contribution
**Age of the child (months)**	06–23	Ref.					Ref.				
	24–59	0.096	0.003	−0.005	0.000	0.2	− 0.043	− 0.004	− 0.002	0.000	0.0
**Sex of the child**	Male	Ref.					Ref.				
	Female	0.106	0.002	−0.009	0.000	0.2	0.015	0.001	−0.01	0.000	0.0
**Mother’s education**	No education	Ref.					Ref.				
	Primary	−0.379	− 0.002	− 0.02	0.000	− 0.2	− 0.073	− 0.002	−0.169	0.000	−1.3
	Secondary	− 0.622	−0.008	0.375	−0.003	24.5	−0.160	− 0.013	0.187	− 0.002	12.7
	Higher	−0.983	− 0.003	0.804	− 0.002	20.5	−0.195	− 0.003	0.642	− 0.002	10.7
**Birth order**	1st order	Ref.					Ref.				
	2–3 order	0.090	0.001	−0.076	0.000	0.6	0.038	0.002	−0.11	0.000	1.2
	4–6 order	0.847	0.000	−0.239	0.000	0.0	0.364	0	−0.301	0.000	0.1
**Currently breastfeeding**	No	Ref.					Ref.				
	Yes	0.135	0.004	−0.082	0.000	3.0	−0.010	− 0.002	− 0.081	0.000	− 0.8
**Wealth index**	Poor	Ref.					Ref.				
	Middle	−0.193	−0.001	0.155	0.000	1.3	−0.012	−0.001	0.145	0.000	0.8
	Rich	−0.505	−0.007	0.676	−0.005	40.7	−0.056	− 0.006	0.672	− 0.004	22.4
**Caste**	SC/ST	Ref.					Ref.				
	Non SC/ST	0.173	0	−0.077	0.000	0.2	0.081	0.008	0.100	0.001	−4.3
**Religion**	Hindu	Ref.					Ref.				
	Muslim	0.071	0.001	0.003	0.000	0.0	0.150	0.007	0.022	0.000	−0.8
	Others	−0.049	0.000	0.308	0.000	0.2	0.097	0.000	0.302	0.000	−0.7
**Place of residence**	Urban	Ref.					Ref.				
	Rural	0.071	0.003	−0.168	−0.001	4.7	−0.028	− 0.012	−0.181	0.002	−10.9
**EAG and non-EAG**	EAG and Assam	Ref.					Ref.				
	Non-EAG	0.676	0.032	−0.015	0.000	4.1	−0.718	− 0.061	0.222	− 0.014	70.8
Explained CI					−0.012	100.0				−0.019	100.0
Actual CI					−0.013					−0.029	
Residuals					−0.001					−0.010	

EAG states include Bihar, Jharkhand, Uttar Pradesh, Uttarakhand, Madhya Pradesh, Chhattisgarh, Orissa, Rajasthan and Assam

*Non-EAG states include the rest of Indian states except EAG and Assam

*CI* concentration index, *Ref* Reference, *EAG* empowered action group, *IS* Iron supplement supplementation, *SC/ST* Scheduled Caste/Scheduled Tribe

## Discussion

As it was found in the present paper that the children who do not receive VAS and IS were more prevalent among children aged 24–59 months. This finding was consistent with the previous one [26]. Mother’s educational status played a protective role for VAS and IS for children. Educated mother has better knowledge about the nutritional levels to be attained for their children, and hence their children receive adequate micronutrients [31]. Higher birth order plays a destructive role in VAS and IS, and this finding was in parallel with previous studies. Earlier studies also discussed that children from higher birth order had a higher deficiency of vitamin A and iron as the first-order child receives much attention and higher allocation resources as a comparison to higher-order births [16]. Increase in VAS was witnessed in children who were breastfed, and this finding was consistent which the previous studies which commented that breastfeeding was less common among children with problem of nigh-blindness and Bitot’s spot [32]. Interestingly, the children who did not receive VAS were not significantly associated with the wealth status of the household; however, children from rich households had lower odds for not receiving IS than children from a poor household. The finding was consistent with the previous one, which argued that iron deficiency was high among households with poor food security and low-income status [33].

It was found that children from rural areas had a higher prevalence for not receiving VAS and IS; however, surprisingly, for adjusted estimates, children from rural areas had lower odds for not receiving VAS. However, it was cited that children from rural areas had a higher prevalence of micronutrient deficiency [34]. In 2005–06 it was found that odds for not receiving VAS and IS was higher among children from non-EAG states; however, in 2015–16, the results revealed an opposite situation. The result was interesting and needed further research to look into the possible reasons.

The contribution of maternal education towards explaining SES related inequality for both VAS and IS was high in 2005–06, but the contribution declined in 2015–16. However, in both time periods, the contribution was positive and significantly high. The result was consistent with the previous study that children of educated mothers’ had health advantageous due to their higher socio-economic status [35]. Moreover, with the recent advancement of the Integrated Child Development Scheme (ICDS), even women with no education are getting aware of optimum micronutrient intake among their child [36]. The contribution of household wealth status was also a significant contributor towards explaining the SES related inequality for VAS and IS. The result was consistent with previous studies that wealth status explains a large SES related gap for micro-nutrient intake status among children [37]. The contribution of the rural place of residence was negative for VAS in 2005–06, and 2015–16 as children from rural areas had a lower likelihood for VAS and children from rural areas too belonged to poor SES hence producing negative contribution [38]. The contributing also declined due to better nutritional food received by children in rural areas because of the proper implementation of ICDS programmes in rural India [39]. Surprisingly, in 2005–06 it was found that non-EAG states were having a higher likelihood of VAS and IS; however, the situation got reverse in 2015–16. Moreover, children from non-EAG states were disproportionally poor (negative concentration index) in 2005–06, and their situation was better (positive concentration index) in 2015–16. The huge change in contribution was due to a significant change in the value of the concentration index in 2005–06 and 2015–16 [38].

The study had some limitations too. For instance, VAS and IS were self-reported and not clinically tested. Moreover, the study was cross-sectional, so it cannot capture the true picture of change in VAS and IS at the individual level. However, beyond all the limitation, the study provides a broad glimpse of increasing VAS and IS among poor children.

## Conclusion

It was revealed that children who did not receive VAS and IS got more concentrated into the lower socio-economic status from 2005 to 06 to 2015. Mother’s educational status, birth order, breastfeeding status and residential status were the factors that were significantly associated with VAS and IS. Additionally, it was found that maternal education, wealth status and EAG and non-EAG status of states contributed most towards explaining SES related inequality for VAS and IS among children in India. Therefore there is a need to focus on children from lower socio-economic strata who are more prone to deficiency of VAS and IS. Schemes like ICDS would play a significant role in reducing SES related gap for micro-nutrient supplementation among children in India. Proper implementation of ICDS will reduce the gap between rich and poor children regarding micro-nutrient supplementation.

## Data Availability

The study utilizes secondary sources of data that are freely available in the public domain through https://dhsprogram.com/methodology/survey/survey-display-355.cfm. Those who wish to access the data may register at the above link and thereafter can download the required data free of cost.

## Abbreviations

IS: Iron supplementation
VAS: Vitamin A supplementation
ID: Iron deficiency
VAD: Vitamin A deficiency
SC/ST: Scheduled Caste/Scheduled Tribe
OR: Odds ratio
CI: Concentration index
EAG: Empowered Action Group
SES: Socio-economic status
CC: Concentration curve

## References

[1] Mason JB, Dalmiya N (2001). The micronutrient report.

[2] WHO (2016). Guideline daily iron supplementation in infants and children.

[3] Uncief (2018). Micronutrients | Nutrition |.

[4] Thompson J, Biggs BA, Pasricha SR. Effects of daily iron supplementation in 2- to 5-year-old children: systematic review and meta-analysis. Pediatrics. 2013. 10.1542/peds.2012-2256 23478873

[5] Kotecha P (2011). Nutritional anemia in young children with focus on Asia and India. Indian J Community Med.

[6] Wong C. Iron deficiency anaemia. Paediatr Child Health. 2017.

[7] Kim JY, Shin S, Han K, Lee KC, Kim JH, Choi YS, Kim DH, Nam GE, Yeo HD, Lee HG, Ko BJ (2014). Relationship between socioeconomic status and anemia prevalence in adolescent girls based on the fourth and fifth Korea National Health and Nutrition Examination Surveys. Eur J Clin Nutr.

[8] Godwin JG (2019). Risk factors and socioeconomic indicators of iron deficiency anemia in children under 5 years of age in Rural Imo State, Nigeria.

[9] Bharati S, Pal M, Chakrabarty S, Bharati P (2015). Socioeconomic determinants of iron-deficiency anemia among children aged 6 to 59 months in India. Asia Pac J Public Health.

[10] Sharma H, Singh SK, Srivastava S. Socio-economic inequality and spatial heterogeneity in anaemia among children in India: evidence from NFHS-4 (2015–16). Clin Epidemiol Glob Health. 2020;0(0).

[11] Bell RAF. Nelson. Textbook of pediatrics. Arch Dis Child. 1997.

[12] Nazari M, Mohammadnejad E, Dalvand S, Gheshlagh RG. Prevalence of iron deficiency anemia in Iranian children under 6 years of age: a systematic review and meta-analysis. J Blood Med. 2019. 10.2147/JBM.S196102 PMC6499484 31118852

[13] Ezzati M (2006). Comparative quantification of mortality and burden of disease attributable to selected risk factors.

[14] Haidar J. Common micronutrient deficiencies among food aid beneficiaries: evidence from refugees in Ethiopia. Ethiop J Health Dev. 2012.

[15] Stevens GA, et al. Trends and mortality effects of vitamin A deficiency in children in 138 low-income and middle-income countries between 1991 and 2013: a pooled analysis of population-based surveys. Lancet Glob Health. 2015. 10.1016/S2214-109X(15)00039-X 26275329

[16] Chaudhry AB, Hajat S, Rizkallah N, Abu-Rub A. Risk factors for vitamin A and D deficiencies among children under-five in the state of Palestine. Confl Heal. 2018. 10.1186/s13031-018-0148-y PMC5880068 29619077

[17] Tariku A, Fekadu A, Ferede AT, Mekonnen Abebe S, Adane AA. Vitamin-A deficiency and its determinants among pre-school children: a community based cross-sectional study in Ethiopia. BMC Res Notes. 2016. 10.1186/s13104-016-2134-z PMC4920990 27342570

[18] Chen MH, et al. Association between psychiatric disorders and iron deficiency anemia among children and adolescents: a nationwide population-based study. BMC Psychiatry. 2013. 10.1186/1471-244X-13-161 PMC3680022 23735056

[19] Nestel P, Melara A, Rosado J, Mora JO. Vitamin A deficiency and anemia among children 12–71 months old in Honduras. Rev Panam Salud Publica/Pan Am J Public Health. 1999. 10.1590/s1020-49891999000600005 10446513

[20] Lerner V, Miodownik C (2012). Vitamin D deficiency.

[21] Díaz JR, De las Cagigas A, Rodríguez R. Micronutrient deficiencies in developing and affluent countries. Eur J Clin Nutr. 2003. 10.1038/sj.ejcn.1601820 12947458

[22] Sahile Z, et al. Prevalence of vitamin A deficiency among preschool children in Ethiopia: a systematic review and meta-analysis. Biomed Res Int. 2020. 10.1155/2020/8032894 PMC7073500 32258145

[23] UNICEF. Vitamin & mineral deficiency. Micronutrient Initiat. Available: https://www.unicef.org/media/files/vmd.pdf. Accessed 04 Mar 2021.

[24] WHO (2014). Biennium report.

[25] IIPS and ICF (2017). National family health survey (NFHS-4), 2015–16: India.

[26] Deshpande A. Caste at birth? Redefining disparity in India. Rev Dev Econ. 2001.

[27] Arokiasamy P, Gautam A. Neonatal mortality in the empowered action group states of India: trends and determinants. J Biosoc Sci. 2008. 10.1017/S0021932007002623 18093346

[28] Osborne J, King JE (2011). Binary logistic regression. Best practices in quantitative methods.

[29] Wagstaff A. Socioeconomic inequalities in child mortality: comparisons across nine developing countries. Bull World Health Organ. 2000. PMC2560599 10686730

[30] Singh SK, Srivastava S, Chauhan S (2020). Inequality in child undernutrition among urban population in India: a decomposition analysis. BMC Public Health.

[31] Harding KL, Aguayo VM, Masters WA, Webb P (2018). Education and micronutrient deficiencies: an ecological study exploring interactions between women’s schooling and children’s micronutrient status. BMC Public Health.

[32] Mahalanabis D (1991). Breast feeding and vitamin A deficiency among children attending a diarrhoea treatment centre in Bangladesh: a case-control study. Br Med J.

[33] Bayoumi I (2018). Iron deficiency among low income Canadian toddlers: a cross-sectional feasibility study in a Community Health Centre and non-Community Health Centre sites. BMC Fam Pract.

[34] Wong AYS, Chan EW, Chui CSL, Sutcliffe AG, Wong ICK (2013). The phenomenon of micronutrient deficiency among children in China: a systematic review of the literature. Public Health Nutr.

[35] WHO (2019). WHO | daily iron supplementation in children 24–59 months of age.

[36] Mittal N, Meenakshi JV. Does the ICDS improve children’s diets? Some evidence from rural Bihar. J Dev Stud. 2019.

[37] Agrawal S, Agrawal P. Vitamin A supplementation among children in India: does their socioeconomic status and the economic and social development status of their state of residence make a difference? Int J Med Public Health. 2013. 10.4103/2230-8598.109322 PMC4340548 25729705

[38] Yiengprugsawan V, Lim LLY, Carmichael GA, Sidorenko A, Sleigh AC. Measuring and decomposing inequity in self-reported morbidity and self-assessed health in Thailand. Int J Equity Health. 2007;6(1). 10.1186/1475-9276-6-23. 10.1186/1475-9276-6-23 PMC2242789 18088434

[39] Alim F, Jahan F. Assessment of nutritional status of rural Anganwadi children of Aligarh under the ICDS (integrated child development services) and rural health. Stud Home Community Sci. 2012.

